# Letter to the editor: Reply

**DOI:** 10.1186/s12871-021-01306-9

**Published:** 2021-03-17

**Authors:** Jung-Woo Shim, Min Suk Chae

**Affiliations:** Department of Anesthesiology and Pain Medicine, Seoul St. Mary’s Hospital, College of Medicine, The Catholic University of Korea, 222, Banpo-daero, Seocho-gu, Seoul, 06591 Republic of Korea

***Dear Editor:***

We appreciate the valuable comments on our submitted article [1] provided by Löffel et al. We compared the analgesic outcomes on postoperative day (POD) 1 in patients undergoing robot-assisted laparoscopic prostatectomy (RALP) who received a rectus sheath block (RSB) or an intrathecal morphine and bupivacaine block (ITMB) with those of patients who received intravenous patient-controlled analgesia (IV-PCA) alone. Below are our responses to each comment.

In our hospital, we routinely provide a postoperative analgesic measure for a patient who underwent RALP. Patients can choose between ITMB with IV-PCA, RSB with IV-PCA, and IV-PCA only. Informed consent was obtained after detailed information about the advantages and disadvantages of each measure was discussed. Each analgesic protocol was administered to approximately 30% of patients, a finding that may be related to the distribution of the study population into three groups of 30 patients. Because we wanted to make sure that ITMB was superior to other pain control methods performed in our institution, we designed our study to investigate the severity of postoperative pain, as a primary outcome, according to the pain-relief methods. There is a risk of respiratory depression with ITMB. We are always concerned about this potentially life-threatening risk. This is the reason why we began the current study. We wanted to find out if RSB with IV-PCA has a comparable pain control efficacy to ITMB with IV-PCA.

The patients undergoing prostate surgery in our study, including RALP, were elderly (median [IQR] age = 65 [62–71] years) [1]. Surgeons and physicians typically have clinical concerns regarding postoperative desaturation due to atelectasis or residual effects of analgesic or anesthetic drugs, outside of our trial. In the ward, the patients’ saturation was routinely monitored for 24 h postoperatively. During the study period, there were no episodes of desaturation related to respiratory depression thanks to meticulous monitoring and/or decreased opioid infusion, including intrathecal morphine (0.2 mg) [2].

The minimum sample size was based on the difference in cumulative IV-PCA drug consumption on POD 1 between patients who received RSB and those who received ITMB, calculated using preliminary and retrospective data from electronic medical records. Mean cumulative IV-PCA drug consumption on POD 1 by patients who received RSB (*n* = 10) and those who received ITMB (*n* = 10) were 47.1 and 29.1 mL, respectively. The standard deviation (SD) among the 20 patients was 23.6 mL. Therefore, a minimum sample size of 27 patients/group was required to afford an α value of 0.05 and a power of 0.8. We recruited 30 patients for each group assuming a dropout rate of 10% (these are demonstrated in the Statistical analysis section of our article) [1].

Our study was limited in that patients were not randomly allocated, despite the use of comparable groups. A randomized setting was considered but rejected due to ethical concerns that IV-PCA alone may provide insufficient pain relief compared with the other two analgesic regimens. Therefore, it was not possible to determine whether the analgesic results were solely related to the pain relief regimens. However, our study suggests that ITMB, as an analgesic protocol that targets both parietal and visceral pain, may contribute superior pain relief and better patient perception in terms of early postoperative recovery following RALP, which is a more advanced and less invasive surgical technique. Additional randomized studies are required to validate our analgesic results in the field of RALP.

## Data Availability

Not applicable.

## Abbreviations

POD: Postoperative day
RALP: Robot-assisted laparoscopic prostatectomy
RSB: Rectus sheath block
ITMB: Intrathecal morphine and bupivacaine block
IV-PCA: Intravenous patient-controlled analgesia
